# Predictors of emotional distress in uveal melanoma survivors: a systematic review

**DOI:** 10.1038/s41433-022-02193-1

**Published:** 2022-08-08

**Authors:** Cari Davies, Stephen Lloyd Brown, Peter Fisher, Laura Hope-Stone, Debra Fisher, Andrew Morgan, Mary Gemma Cherry

**Affiliations:** 1 Primary Care and Mental Health, University of Liverpool, Liverpool, UK; 2 Clinical Health Psychology Service, The Royal Liverpool University Hospital, Liverpool University Hospitals Foundation Trust, Liverpool, UK; 3 School of Psychology, University of Plymouth, Plymouth, UK; 4 Liverpool Ocular Oncology Centre, The Royal Liverpool University Hospital, Liverpool University Hospitals Foundation Trust, Liverpool, UK

**Keywords:** Cancer, Quality of life

## Abstract

Uveal melanoma (UM) survivors can experience significant emotional distress, although the factors underpinning this are poorly understood. Systematic reviews of distress in UM only include cross-sectional studies, thereby limiting our understanding of causal factors. This review identified prospective clinical, demographic, social and psychological predictors of distress in UM survivors. A systematic search of the literature for English language prospective studies was conducted. Thirteen papers, reporting data from seven unique datasets were included in a narrative synthesis of the results. Younger age (3 studies from 3 datasets), physical health (including visual impairment, ocular symptoms, and other UM-related factors; 3 studies from 3 datasets), and psychological factors (mainly baseline distress; 3 studies from 3 datasets and worry about recurrence; 2 studies from 2 datasets), significantly predicted distress. There was no consistent evidence for other demographic, clinical or social variables (significant in <50% of datasets). Generally, the quality of the papers was adequate. However, attrition rates were high or not reported in over half of the included studies. The findings of this review emphasise the importance of attempts to prevent and recognise distress immediately post-diagnosis of UM. Particular focus should be given to younger patients, those with physical and psychological health difficulties at the time of diagnosis, and those who develop adverse treatment symptoms during survivorship. More research into potential social and psychological variables and their role in predicting distress in survivors is recommended.

## Abstract (zh)

葡萄膜黑色素瘤 (UM) 的幸存者可能会经历严重的情绪困扰, 尽管支持这一论点的因素尚不清楚。对UM患者情绪低落的系统评价仅包括横断面研究, 因此限制了我们对因果因素的理解。本综述确定了UM幸存者心理痛苦的临床、人口统计学、社会和心理的前瞻性预测因子。

本文系统性检索了英文前瞻性研究文献。对13篇论文来自7个独特数据集的数据进行了综合性报道。较年轻 (3个数据集的3项研究) 、身体健康 (包括视力障碍、眼部症状和其他UM相关因素；3个数据集的3项研究) 和心理因素 (主要是痛苦基线值；3个数据集的3项研究和复发的顾虑；2个数据集的2项研究) 是预测患者心理痛苦的显著性预测指标。其他人口统计学、临床或社会变量没有一致的证据 (在数据集中的显著性<50) 。纳入的所有论文质量达标。然而, 在超过一半的研究中, 患者失访率率较高或没有报告。本综述的研究结果强调了在确诊UM后立即预防和识别UM心理痛苦的重要性。我们应特别关注年轻患者、诊断时生理和心理健康障碍的患者以及在生存期间出现不良症状的患者。本文建议对潜在的社会和心理变量及其在预测幸存者痛苦中的作用进行更多研究。

## Introduction

The prevalence of clinically significant levels of emotional distress in people with cancer is between 10 and 20%, which exceeds population levels at 5–7% [1, 2]. Emotional distress incorporates a range of emotional and cognitive responses defined as any negative mood state, including anxiety, depression, trauma symptoms and global distress [3]. Higher levels of emotional distress can result in poorer adherence to treatment [4] and increased levels of pain and fatigue [5]. The processes and predictors underpinning the development and maintenance of elevated emotional distress in cancer are poorly understood [6]. However, specific factors related to the type of cancer seem to be important when considering why some people experience elevated levels of distress that remain over time [7].

Currently, few predictors of distress factors have been identified in uveal melanoma (UM) survivors. UM is the most common primary eye cancer in adults, affecting 5.1 people per million [8], most commonly found among Caucasian (94.7%), followed by Hispanic (3.9%), African American (0.5%) Asian/Pacific Islander (0.7%) and Native American (0.2%) adults [9]. Whilst treatments are generally successful [10], treated UM patients show greater emotional distress and poorer quality of life (QoL) than age-matched populations [11]. UM treatments can cause significant morbidity to the eye and vision, and consequent loss of visual function. Treatments that conserve the eye—plaque radiotherapy, proton beam radiotherapy and local resection [12]—can cause complications such as radiation-induced retinopathy, cataracts, retinal detachment, neovascular glaucoma and macular oedema. Fifty-eight percent experience moderate loss of vision [13]. Approximately 35% of patients require enucleation [12]. These patients consequently lose binocular vision, and 50–75% are affected by other adverse outcomes including socket complications and phantom eye syndrome [14–16]. Around 30–39% of survivors report ocular irritation, appearance concerns, headaches and vision-related functional difficulties [17]. In summary, most survivors report concerns about treatment-related side effects following treatment.

Irrespective of treatment type, up to 50% of patients develop metastatic disease, usually of the liver and most between 5 and 10 years following a UM diagnosis [18, 19]. During one study, 64% of participants died, 77% of these from metastatic disease, 3.9% from other known causes and 4.7% from unknown causes [20]. Metastatic disease develops almost exclusively in patients whose tumour cells show deletion of one of the normal two copies of chromosome 3, which is the presence of monosomy 3 (M3) [21]. There is no adjuvant systemic therapy that reduces the risk of developing metastatic disease [22] and, despite advances in treatment, there has been no substantial improvement in survival rates over three decades [8, 9]. However, there is emerging evidence of increased survival rates from Tebentafusp for metastatic disease [23–25]. Regardless, many patients live with the knowledge of metastatic disease and, overall, 15% of these patients die within 1 year [26]. Further, some ocular oncology services offer prognostic testing—cytogenetic analysis of the tumour to predict probable life expectancy [27]. Whilst prognostic testing has no impact on medical decisions around treatment or influence on survival rates [28, 29], many patients may need to decide on a prognostic test that would reveal their probable life expectancy. Irrespective of whether patients undergo prognostic testing, or the testing outcome, patients report uncertainty and distress over the possibility of metastatic disease [14].

Attempts have been made to investigate predictors of emotional distress and QoL in UM. Two recent reviews found that neither prognostic testing nor treatment type, nor their effects, were associated with consistently elevated levels of distress or significant reductions in QoL [11, 30]. Age, gender, physical health, distress at time of diagnosis and other psychosocial factors were not reliable predictors. Included studies were mostly cross-sectional, retrospective, observational or case control designs. These allow for identification of associations between these factors and distress but cannot demonstrate a causal relationship for future emotional distress, a key criterion for causality, or any critical timelines. Prospective studies provide stronger indications of causality and timeline, identifying predictors of persistent distress that can inform the development of prevention strategies and psychological interventions at key timepoints in the trajectory. Several recent studies have used prospective designs to identify demographic factors, treatments, physical symptoms, and social and psychological predictors of distress, but no systematic review of these studies has been conducted. The aim of this review, therefore, was to systematically identify, synthesise and appraise the findings of prospective studies examining predictors of distress in treated UM patients.

## Method

This systematic review was conducted and reported in accordance with the general principles set out by the Preferred Reporting Items for Systematic Reviews and Meta-analysis (PRISMA) Statement [31].

### Search strategy

AMED, CINAHL plus, Medline, PsycINFO and PsycARTICLES were searched from their inception to January 2020, using a combination of search terms relating to emotional distress and UM (see Table 1 for full search terms). The reference list of relevant studies and systematic reviews were searched for additional relevant literature. Forward and backward citation chaining was conducted using Google Scholar. Searches were repeated every 6 months to identify any relevant new publications in line with the Cochrane Handbook, using the last date of the original search as the beginning date for the update. The last update was on 16/07/2021.

**Table 1:** Predictors in uveal melanoma database search strategy (find any of my search terms) 1975–January 2020.

Connector	Search Term	Search field
	‘uveal melanoma’ OR ‘ocular melanoma’ OR ‘ocular tum*’ OR ‘ocular neoplasm’ OR ‘choroidal melanoma’ OR ‘ciliary body’	All fields
AND		
	‘emotional distress’ OR ‘psychological distress’ OR ‘anxiety’ OR ‘depress*’ OR ‘depressive disorder’ OR ‘anxiety disorder’ OR ‘posttraumatic stress’ OR ‘PTSD’ OR ‘psychological morbidity’ OR ‘psych*’, ‘adjustment’ OR ‘emotional adjustment’ OR ‘mood’ OR ‘adjustment disorder’ OR ‘acute stress disorder’ OR ‘fear of recurrence’ OR ‘distress’ OR ‘general distress’ OR ‘quality of life’ OR ‘QoL’ OR ‘health related quality of life’ OR ‘HRQOL’	All fields
AND		
	‘predict*’ OR ‘risk factor*’ OR ‘caus*’ OR ‘vulnerability’ ‘correlat*’ OR ‘associat*’ OR ‘prospective’ OR ‘cross-section*’	All fields
NOT		
	‘adolescent cancer’ OR ‘child* cancer’ OR ‘paed*cancer’ OR ‘palliative’	AB

### Screening and selection

Following de-duplication, titles and abstracts of identified citations were screened. The full texts of potentially relevant papers were then examined. At both stages, screening was done by two reviewers (CD and DF), with the views of the wider research team sought if consensus could not be reached. Studies were eligible for inclusion if they: (1) were prospective peer-reviewed studies; (2) reported data pertaining to the relationship between any clinical, demographic, social or psychological variable(s) and (3) emotional distress, assessed at follow up at least one month later using validated measure(s) and/or subscales of validated measure(s); (4) presented results for adults (aged ≥ 18 years of age) with a diagnosis of UM; and (5) were published in English. Emotional distress was operationalised as any negative mood state, such as anxiety, depression, trauma symptoms and global distress. Studies were excluded if retrospective, cross-sectional or case studies and/or did not report bivariate or multivariate associations between predictor variables and emotional distress.

### Data extraction

Data were independently extracted and tabulated by two reviewers (CD and DF); disagreements were resolved by consensus, with MGC and SB consulted in the case of disagreement. The extracted data were sample and study characteristics, emotional distress measures, predictors (categorised as demographic, clinical, social and psychological) and statistical methods and results. Data were tabulated and analysed narratively due to heterogeneity in predictor variables, measures of distress, study objectives, follow up timing and statistical analysis.

### Data synthesis

Papers reporting data from the same or linked sample(s) were treated as a single study for the purposes of quality assessment and data synthesis. Seven papers from one research group (University of Liverpool, UK) reported linked data derived from the same large dataset (collected April 2008 to December 2019). Three [32–34] analysed the same sub-sample of patients but reported different analyses and predictors. Another paper [35] analysed an overlapping but larger sample (*n* = 703). A proportion of these participants (*n* = 453) were also analysed in Brown et al. [36]. Two [37, 38] analysed a yet larger sub-sample, each reporting different predictors and analyses from 1993 to 2013. In the narrative summary, where data overlap was present in two or more papers, we used the paper with the largest sample, longest recruitment period and relevant clinical and demographic covariates. On occasions, we chose to cite a paper with overlapping participants because it provided additional fine-grained analysis (e.g., better statistical control) that contributed to the narrative. This is made clear in the text where necessary. Overlapping datasets are made clear in the text. Tables contain complete information for each study and readers should be aware that overlapping data are presented. Dataset overlap between studies caused homogeneity in the reviewed papers and increased the probability of sampling error and bias attributable which was considered in reporting the findings to local context.

### Risk of bias

The methodological quality of the included studies was assessed by CD and cross-checked by LHS, using the Newcastle-Ottawa Scale [39] for cohort studies, which assesses the selection of the sample, comparability of groups and outcome, the items relating to comparability of control groups were removed. For selection of groups, samples were considered representative if participants were predominately Caucasian and aged over 50 years, with samples comprising equivalent numbers of males and females and similar frequency of radiotherapy vs. enucleation [9] and varied tumour size with uveal melanoma (ciliary body or choroidal). Ascertainment of exposure was dependent on how the presence of uveal melanoma and associated variables were identified. For outcome, assessment of outcome was considered adequate if validated measures were used. Follow up length of >1 month were adequate for effects to occur. Finally, the adequacy of follow up was defined as small number lost at follow up, 20% (small) 30% (moderate) and 40% (high).

## Results

**Fig. 1:**
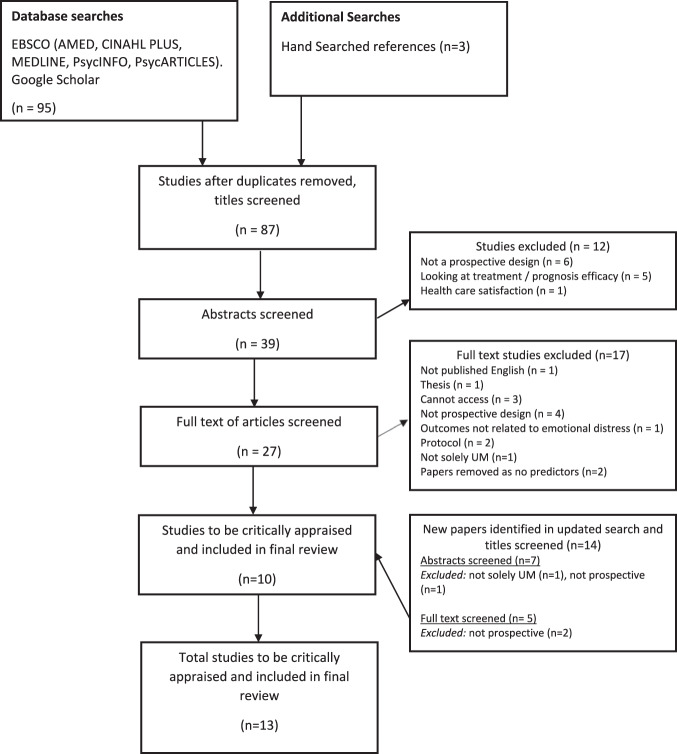
PRISMA flowchart. PRISMA diagram showing the screening process for the included studies.

The electronic and hand searches identified 98 citations, resulting in 90 citations following removal of duplicates. The full-text of 30 papers was obtained and screened against the eligibility criteria. In the updated searches, an additional 14 papers were found, three of which were relevant and included (see Fig. 1). Thirteen papers, reporting data from seven unique datasets, were included in the review. The selection process is shown in Fig. 1.

### Overview of included studies

The characteristics of the studies are shown in Table 2. Thirteen papers, reporting data from seven unique datasets, were included. Eleven studies were conducted in Europe (UK (*n* = 7), Germany (*n* = 2), Netherlands (*n* = 1) and Sweden (*n* = 1), and two in the USA. Participants’ median age ranged from 62 to 64 years. Only one study [40] did not include participants treated with some form of radiotherapy, and only two did not include participants who were enucleated [40, 41]. Five papers used pre-treatment as a baseline, which varied from first admission (*n* = 3) [41–43], second admission (*n* = 1) [44] or immediately before treatment (*n* = 1) [40]. One paper considered post-treatment, 6 weeks after surgery, as baseline [45]. The eight remaining papers used 6 months post-treatment as the baseline [32–38].

**Table 2:** Study characteristics.

Author and year	Demographics mean or median age (years) SD and (range)	Sample size	Treatment, *n* (%)	Tumour characteristics	Attrition (%)	Time since treatment (weeks/months)	Follow-up Duration (months)	Country
Brandberg et al. [42]	51.51% female female x̅ age = 63 (27–88) male x̅ age = 64 (31–87) SD (±) = NR	T1 = 99 T2 = 85 T3 = 78	Enucleation, 49 (49) Ruthenium plaque radiotherapy, 50(51)	Origin/location: Ciliary body (*n* = 6) Size: 4–22 mm diameter and 2–20 mm thickness. TNM: NR	NR	0 (First admission)	T2 = 2 T3 = 12	Sweden
Brown et al. [32]	49% female age = NR	T1 = 411 T2 = 325 T3 = 261	Enucleation 66 (25.3) Brachytherapy: ruthenium plaque 116 (44.4) Proton beam radiotherapy 49 (18.8) Local resection 9 (3.4) Endoresection 12 (4.6) Photo dynamic therapy 7 (2.7) Transpupillary thermal therapy 2 (0.8)	Origin/location: choroid and ciliary body Size: NR TNM: NR	36.5%	6	T2 = 12 T3 = 24	UK
Brown et al. [35]	48.4% female x age = 69.03 (±12.12)	T1 = 814 T2 = 773 T3 = 706 T4 = 619 T5 = 557 T6 = 453	Plaque radiotherapy 327 (46.2) Proton beam radiotherapy 167 (23.6) Enucleation 155 (21.9) Resection 34 (4.8) Other 25 (3.5)	Origin/location: choroid and ciliary body Size: NR TNM: NR	NR	6	T2 = 12 T3 = 24 T4 = 36 T5 = 48 T6 = 60	UK
Brown et al. [36]	47.0% female x age = 69.48 (±11.57)	T1 = 814 T2 = 773 T3 = 706 T4 = 619 T5 = 557 T6 = 453	Plaque radiotherapy 219 (48.3) Proton beam radiotherapy 99 (21.9) Enucleation 92 (20.3) Resection 26 (5.7) Other 17 (3.8)	Origin/location: choroid and ciliary body Size: NR TNM: NR	44.3%	6	T2 = 12 T3 = 24 T4 = 36 T5 = 48 T6 = 60	UK
Damato et al. [38]	48.30% female female x̅ = 62.5 (21.6–94.4) male x̅ = 62 (25.9–91.7) SD (±) = NR	N = 1596	Ruthieum plaque therapy or proton beam therapy, 1154 (72.3) Enucleation, 442 (27.7)	Origin/location: choroid & ciliary body Size: NR TNM: NR	NR	6	T2 = 12 T3 = 24 (Up to 10 years)	UK
Damato et al. [37]	48.30% female age = NR	*N* = 1569	Ruthieum plaque therapy or proton beam therapy, 1154 (72.3) Enucleation, 442 (27.7)	Origin/location: choroid and ciliary body Size: NR TNM: NR	NR	6	T2 = 12 T3 = 24 (Up to 10 years)	UK
Hope-Stone et al. [33]	51.30% female age = NR	T1 = 194 T2 = 155 T3 = 132	Enucleation 66, (57.4) Proton beam therapy 49 (42.6)	Origin/location: choroid and ciliary body Size: Height 5.59 mm (SD = 3.74) 0.6–14.8 Diameter 13.04 mm (SD = 4.56), 0.99–20.5 TNM: NR	40.7%	6	T2 = 12 T3 = 24	UK
Hope-Stone et al. [34]	47% female x̅ age = 64 (23–94) SD (±) = NR	*T* = 411 *T* = 325 *T* = 291	Ruthenium plaque radiotherapy 174, (28.5) Proton beam radiotherapy 72 (17.5) Trans-scleral local resection 15 (3.6) Endoresection 21 (5.1) Enucleation 117 (28.5) Photo dynamic therapy 10, (2.4), Transpupillary thermal therapy 2, (0.5).	Origin/location: choroid (381) and ciliary body (30) Size: NR TNM: NR	NR	6	T2 = 12 T3 = 24	UK
Klingenstein [41]	47.25% female x̅ age = 63 (27–86) SD (±) = NR	*N* = 91	Cyberknife radiosurgery	Origin/location: Uveal Melanoma with - ciliary body (5) - peripapillary region (15) - posterior pole (14) - midperiphery (34) - periphery (23) Size: NR TNM: T1 = 12 T2 = 29 T3 = 45	NR	0 (First admission)	T2 = 3 T3 = 15	Germany
Lieb et al. [43]	33.3% female Genetic Test group: 57.68 (±12.03) 51.8% female Observational: 63.08 (±11.4)	T1 = 186 T2 = 163 T3 = 154 T4 = 150 T5 = 142	Enucleation (test: 23, observational: 8), Brachytherapy (test: 25, observational: 90), Endo-resection with adjuvant brachytherapy (test: 7, observational: 3), proton therapy (test: 6, observational: 7).	Origin/location: diagnosis of UM with possibility of tumour sample removal. Size: NR TNM: NR	NR	0 (First admission)	T2 = 6–12 wks T3 = 6 T4 = 12 T5=	Germany
Melia et al. [45]	47.36% female x̅ age = 62 (29–87) SD (±) = NR	T1 = 206 T2 = NR T3 = NR T4 = 178 T5 = 168 T6 = 60	Iodine 125 brachytherapy 103 (50) Enucleation 103 (50)	Origin/location: unilateral choroidal Size: 2.5–10.0 mm in apical height and no more than 16.0 mm in basal diameter. TNM: NR	9%	6 weeks	T2 = 12 T3 = 24 T4 = 36 T5 = 48 T6 = 60	USA
Schuermeyer et al. [40]	44% female x̅ age = 60.7 SD = (±14.8)	T1 = 96 T2 = 68 (HADS)/65 (DRS) T3 = 79 (HADS)/77 (DRS)	-	Origin/location: NR Size: NR TNM: NR	NR	0 (Immediately pre treatment)	T2 = 3 T3 = 12	USA
Van Beek et al. [44]	46.90% female x̅ age = 65 (33–89) SD (±) = NR	T1 = 111 T2 = 113 T3 = 110 T4 = 104 T5 = 75 T6 = 61 T7 = 53	Fractionated stereotactic radiotherapy 66, (58) Enucleation 47 (42)	Origin/location: N/A Size (mm): fRST: mean largest tumour diameter 67.0 (12.1 SD) mean tumour thickness 6.0 (2.5) enucleation: mean largest tumour diameter 63.3 (12.6) mean tumour thickness 7.5 (4.3) TNM: T1 = 29 T2 = 35 T3 = 40 T4 = 9	NR	0 (Second admission)	T2 = 2 T3 = 6 T4 = 12 T5 = 24 T6 = 36 T7 = 48	Netherlands

Clinical and medical records and self-report questionnaires were used to assess clinical, demographic, social and psychological predictors across all studies. Ten papers assessed depression and anxiety, and one additional paper assessed anxiety only. Two papers considered general distress and one fear of progression. Twelve papers assessed emotional QoL. See Table 3 for full description of self-report questionnaires.

**Table 3:** Validated questionnaires of emotional distress in eligible studies.

Dependent Variable	Measure	Used by
Depression	HADS-D	[32, 34–38, 40, 42, 43, 45]
Anxiety	HADS-A	[32, 34–38, 40, 42, 43, 45]
	STAI	[44]
	FoP-Q	[43]
Distress	DT	[43]
	IES	[42, 44]
Quality of Life (subscales of emotional distress)	EORTC QLQ-C30 (emotional functioning; WREC)	[32, 33, 35–38, 42, 44]
	SF-36 (mental health, role emotional)	[45]
	FACT-G (emotional)	[34, 35, 37, 38]
	SF-12 (emotional, mental health)	[41, 43]

*HADS* Hospital Anxiety and Depression Scale, *STAI* The State-Trait Anxiety Inventory, *FoP-Q* The Fear of Progression Questionnaire, *DT* Distress Thermometer, *IES* Impact of Events Scale, *EORTC QLQ-OPT 30* European Organisation for Research and Treatment for Cancer Opthalamic Quality of Life Module, *WREC* Worry about Recurrent Disease, *SF-36* The 36-Item Short Form Survey, *FACT-G* Functional Assessment of Cancer Therapy: General, *SF-12* The 12-Item Short Form Health Survey.

### Risk of bias

The risk of bias for the 13 included papers is presented in Table 4. Seven papers adequately reported sample characteristics and were reflective of the average community sample as described in line with Mahendraraj et al. [9]. From the remaining six papers, half were partially representative and others did not report sample characteristics. All papers used clinical/medical records or a combination of clinical records and self-report questionnaires to determine ascertainment of exposure. All papers used validated measures or subscales of validated measures to assess emotional distress. All papers had reported adequate follow up of at least 1 month or more. Only two papers reported more than 80% retention over the course of the prospective data collection [42, 45]. One paper reported between 70 and 80% retention [34] and another paper between 60 and 70% [32]. Two papers reported attrition rates under 60% [33, 36]. Seven papers did not report their attrition rates [37, 38, 41] and four did not report percentages but gave some description of those lost and provided number of participants lost [35, 40, 43, 44].

**Table 4:** Risk of bias using adapted Newcastle–Ottawa Quality Assessment Scale: Cohort Studies.

	Selection		Outcome		
Study	Representative of the exposed cohort?	Ascertainment of exposure?	Assessment of outcome?	Was follow-up long enough for outcomes to occur?	Adequacy of follow up cohorts?
Brandberg et al. [42]	Yes - Different tumour sizes, treatments, equal men and women, age range	Yes - Clinical records	Yes - Validated measures of emotional distress	Yes – 2 months	Yes - subjects lost to follow up unlikely to introduce bias – small number lost (86%) and description provided of those lost.
Brown et al. [32]	Different tumour types and treatment, male and female. Age not reported.	Yes - Clinical records and self-report	Yes - Validated measures of emotional distress	Yes –12 months	Yes - subjects lost to follow up unlikely to introduce bias – moderate number lost (63.5%) and description provided of those lost.
Brown et al. [35]	Choroid and ciliary body, equal men and women, age limited to 69 (+−12), No description of tumour type or size.	Yes - Clinical records and self-report	Yes - Validated measures of emotional distress	Yes – 12 months	Partially - no percentage reported, some lost at follow up, some description of those lost
Brown et al. [36]	Unclear - Ciliary body and choroidal melanoma, treatment type varied. No description of age, gender or tumour size.	Yes - Clinical records and self-report	Yes - Validated measures of emotional distress	Yes – 12 months	No - large number lost at follow up (55.7%), no description of those lost
Damato et al. [38]	Yes -, Different size and tumour characteristics (choroidal only), varying treatment, male and female and age.	Yes - Clinical records and self-report	Yes - Validated measures of emotional distress	Yes – 12 months	No description or percentage.
Damato et al. [37]	Yes - Different size and tumour characteristics (choroidal only), varying treatment, male and female and age	Yes - Clinical records	Yes - Validated measures of emotional distress	Yes – 12 months	No description or percentage.
Hope-Stone et al. [33]	Yes - Different tumour characteristics, treatment, male and female, age range 54–70 (average age for UM 65)	Yes - Clinical records and self-report	Partially- Subscale for worry from a complete validated measure used	Yes – 12 months	Partially - high number of subjects lost to follow up, (59.3 %), no description
Hope-Stone et al. [34]	Yes - Age, gender, tumour type, treatment	Yes, Clinical records and self-report	Yes - Validated measures of emotional distress	Yes – 12 months	Yes - Subjects lost to follow up unlikely to introduce bias – moderate number lost (74.2%) and description provided of those lost
Klingenstein et al. [41]	Partially - Somewhat representative, one treatment modality, age range accurate, male and female, tumour types, not sizes	Yes - Clinical records	Yes - Validated measures of emotional distress	Yes – 3 months	No description or percentage.
Lieb et al. [43]	Yes - treatment modality, age range accurate, male female, tumour types and sizes	Yes - Clinical records and self-report	Yes - validated measures of emotional distress	Yes – 6 weeks	Partially - Subjects lost to follow up unlikely to introduce bias, small number lost, no percentage reported, some description of those lost
Melia – COMS [45]	Partially - Only unilateral choroidal melanoma, accurate age, gender, tumour size and treatment	Yes - Clinical records	Yes - Validated measures of emotional distress	Yes – 6 months	Yes - Subjects lost to follow up unlikely to introduce bias – small number lost (91%) and some description provided of those lost
Schuermeyer et al. [40]	Partially - Somewhat representative, men and women, age limited to 60 (+−15), no description of tumour type size	Yes - Clinical records	Yes - Validated measures of emotional distress	Yes – 3 months	Partially - Subjects lost to follow up no percentage reported but moderate proportion from sample, some description including number.
Van Beek et al. [44]	Yes - Equal men and women, age range, treatment type, tumour size	Yes - Clinical records	Yes - Validated measures of emotional distress	Yes – 6 months	Partially - Subjects lost to follow up no percentage reported but small proportion from sample, description provided of those lost.

### Predictors of emotional distress

Table 5 summarises the study design and findings, grouped by type of distress.

**Table 5:** Summary of study design and significant findings from included papers, grouped by dependent variable.

Author and date	Analyses	Follow up periods	Dependent Variable/s	Psychosocial	Non-psychosocial	Significant predictors
DV – DEPRESSION						
Brandberg et al. [42]	Mixed model ANOVA	First admission, 2 and 12 months	HADS-D		Treatment modality (plaque/enucleation)	No between group differences were found when depression was treated as a continuum or when clinical cut-offs were used.
Brown et al. [32] ^a^[linked sample]	Hierarchical logistic regression (controlling anxiety and 6 and 12 months) and mediation analysis.	6, 12 and 24 months	HADS-D	Worry about cancer recurrence Anxiety Depression	Visual and ocular symptoms, visual function problems Post treatment symptoms, Functional problems, Chromosome 3 status (M3 versus others), Gender, Age	24 months Block 1 • 6 month (OR = 12.06) and 12 month (OR = 9.94) depression or anxiety caseness Block 2 (6 month) • Headache (OR = 2.32) • Functional problems (OR = 1.16) • Worry about recurrent disease (OR = 0.71) Block 3 No significant predictors *Non significant* Age, gender, ocular symptoms, visual impairment, M3
Brown et al. [35] ^a^[linked sample]	Growth curve modelling to estimate intercepts and slopes. Intercepts and slopes predicted by prognostic variable (M3, D3, not tested, test failed)	6, 12, 24, 36, 48 and 60 months	HADS-D		Chromosome 3 status, Prognostic test	M3 patients had higher depression intercepts than D3 or not tested. No difference between slopes. Thus, M3 predicts consistently higher depression than D3 or not tested. D3 did not differ from not tested.
Brown et al. [36] ^a^[linked sample]	Cross-lagged analysis examining mediation of relationship between concerns about symptoms and depression by worry about cancer recurrence	6, 12, 24, 36, 48 and 60 months	HADS-D	Anxiety Depression Worry about cancer recurrence	Concerns about symptoms and function (linear combination of Visual and ocular symptoms, visual function problems), Chromosome 3 status (M3 versus others), Age Gender Treatment	12 months 12 month depression predicted by female gender. 36 months+ 36 and 48 month depression predicted by 24 and 36 month concerns and worry about recurrence. In males 12 moth depression predicted 24 month concerns, which predicted 36 month depression. In females, 24 month depression predicted 34 month concerns which then predicted 36 month concerns, which predicted 48-month depression *Non-significant* M3, age, treatment, anxiety
Damato et al. [37] ^a^[linked sample]	Bootstrapped t-test for six month follow-up. Multilevel regression for linear trend over all observations Linear regression	6 months and annual observation for 10 years	HADS-D		Treatment modality	6 months Radiotherapy patients less likely to experience depression (obs. coeff. –0.408). No analysis of treatment differences over time
Damato et al. [38] ^a^[linked sample]	Linear regression predicting depression from treatment controlling treatment, pre-treatment visual acuity, eye laterality, TNM size, melanoma cytomorphology and tumour type. Linear regression predicting depression from age, sex, time between treatment and questionnaire, general health, marital status, employment status and social support.	6 months	HADS-D	Social Support Self report QoL	Treatment modality Gender Age Marital status Employment status Self-reported general health	6 months Enucleation associated with depression (linear regression, 95% confidence interval [CI], 0.17–1.01). Less depression in radiotherapy compared to enucleation (*p* = 0.007). (univariable t-test) Multivariable analysis Poor social support (*p* < 0.001). Employment associated with less depression (*p* < 0.001). Better self-reported general health associated with less depression (*p* < 0.001). *Non-significant* Age, gender, marital status
Hope-Stone et al. [34] ^a^[linked sample]	Mixed model multivariate ANOVA	6, 12 and 24 months	HADS-D		Treatment modality Prognosis (Monosomy 3) Age Gender	6, 12 and 24 months Monosomy 3 patients more depressed over all three time points (*F* = 6.23, df 1259, *p* < 0.05). *Non-significant* No difference in depression for treatment, age or gender.
Melia et al. [45]	Intention to treat analysis. Markov calculation of transition probabilities between diagnostic categories across timepoints compared using log rank tests. Logistic regression used to estimate ORs at separate timepoints controlling baseline category and other covariates. Intention to treat analysis	6 weeks after surgery, 6, 12, 24, 36, 48 and 60 months	HADS-D		Treatment modality	Probability of diagnoses did not differ across treatments. Depression scores did not differ by treatment.
Lieb et al. [43]	Mixed modelling controlling age, sex and enucleation.	First admission, 6–12 weeks, 6 and 12 months.	HADS-D	Social Support General distress Anxiety Depression QoL: physical QoL: mental Perceived risk Resilience Fear of progression	Prognostic testing Enucleation Graduation Age Gender	12 months Lower resilience: *B* (SE) = 0.074 (0.032), *β* = −0.220, (*p* = 0.023) Higher fear of progression: *B* (SE) = 1.326 (0.508), *β* = 0.224, (*p* = 0.010) Lower physical quality of life: *B* (SE) − 0.067 (0.029), *β* = −0.142, (*p* = 0.022) Higher depression at baseline: *B* (SE) 0.418 (0.090), *β* = 0.432, (*p* < 0.001) *Non-significant* age, sex, graduation, social support, general distress, anxiety, QoL mental, perceived risk, enucleation monosomy group
Schuermeyer et al. [40]	Mixed model 2 sided and Bonferroni adjusted	Immediately pre-treatment, 3 and 12 months	HADS-D	Anxiety Depression Decision Regret	Prognostication group Age Gender	12 months Baseline anxiety (coefficient estimate predicts depression [SE], 0.39 [0.06]; *P* < 0.001) Baseline anxiety and depression interact to predicted an increase in depression. No association between depression over time and decision regret score at baseline, results of prognostication test or entry into adjuvant therapy trial. Gender or prognostic group.
DV – ANXIETY						
Brandberg et al. [42]	Mixed model ANOVA	First admission, 2 and 12 months	HADS-A		Treatment modality (plaque/enucleation)	No between group differences were found when depression was treated as a continuum or when clinical cut-offs were used.
Brown et al. [32] ^a^[linked sample]	Pearson and point-biseral correlation. Hierarchical logistic regression (controlling anxiety and 6 and 12 months) and mediation analysis.	6, 12 and 24 months	HADS-A	Worry about recurrent disease Anxiety Depression	Visual and ocular symptoms, visual function problems, Chromosome 3 status, Gender, Age	24 months Block 1 • 6 month (OR = 9.86) and 12 month (OR = 7.56) depression or anxiety caseness Block 2 (6 month) • Headache (OR = 1.77) • Functional problems (OR = 1.12) • Occular irritation (OR = 1.23) Block 3 (12 month) • Worry about recurrent disease (OR = 1.34) WREC at 12 months mediated prediction of anxiety by 6-month symptoms and functional problems. *Non significant* Age, gender
Brown et al. [35] ^a^[linked sample]	Growth curve modelling to estimate intercepts and slopes. Intercepts and slopes predicted by prognostic variable (M3, D3, not tested, test failed)	6, 12, 24, 36, 48 and 60 months	HADS-A		Chromosome 3 status, Prognostic test	M3 patients had higher anxiety intercepts than D3 or not tested. No difference between slopes. Interpretation is that M3 predicts consistently higher anxiety than D3 or not tested. D3 did not differ from not tested.
Brown et al. [36] ^a^[linked sample]	Pearson correlations, cross-lagged analysis examining mediation of relationship between concerns about symptoms and depression by worry about cancer recurrence	6, 12, 24, 36, 48 and 60 months	HADS-A	Anxiety Depression Worry about cancer recurrence	Concerns about symptoms and function (linear combination of Visual and ocular symptoms, visual function problems), Chromosome 3 status (M3 versus others), Gender, Age	12 months 12 month anxiety predicted by 6 month concerns. and worry about recurrence. 24 month anxiety predicted by worry about recurrence. 36 months 36 month anxiety predicted by 24 month concerns and worry about recurrence. 60 months 60 month anxiety predicted by 48 month worry about recurrence. Prediction of 60 month anxiety by 48 month concerns may be mediated by 48 month worry. In males 12-month anxiety predicted 24 month worry, which then predicted 36 month anxiety *Non-significant* M3, age, gender, anxiety, depression
Damato et al. [37] ^a^[linked sample]	Bootstrapped t-test for six month follow-up. Multilevel regression for linear trend over all observations. Linear regression.	6 months and annual observation for 10 years	HADS-A		Treatment modality	No 6-month differences in anxiety. No analysis of treatment differences over time.
Damato et al. [38] ^a^[linked sample]	Linear regression predicting anxiety from treatment controlling treatment, pretreatment visual acuity, eye laterality, TNM size, melanoma cytomorphology and tumour type. Linear regression predicting anxiety from age, sex, time between treatment and questionnaire, general health, marital status, employment status and social support.	6 months	HADS-A	Social Support Self report QoL	Treatment modality Gender Age Marital status Employment status Self-reported general health	6 months Female sex (*p* < 0.001). SE = 0.19 Chromosome 3 loss (*p* = 0.039) SE = 0.36 Older age reduced anxiety - Younger age (*p* < 0.001). SE = 0.01 Poor social support (*p* < 0.001) SE = 0.07 Better general health reduced anxiety - Poor physical health (*p* < 0.001) SE = 0.09 *Non-significant* No difference between treatment, employment status, marital status for anxiety.
Hope-Stone et al. [34] ^a^[linked sample]	Mixed model multivariate ANOVA.	6, 12, 24 months	HADS-A		Treatment modality Prognosis (Monosomy 3) Age Gender	24 months (6, 12 and 24 months) Younger patients compared to older (*F* = 13.21, df 1259, *p* < 0.01) Female sex compared to male (*F* = 19.75, df 1259, *p* < 0.01) *Non-significant* No difference in anxiety between treatment, and monosomy 3.
Lieb et al. [43]	Mixed modelling controlling age, sex and enucleation.	Hospital admission, discharge, 6–12 weeks, and 6 and 12 months.	HADS-A Fear of progression	Social Support General distress Anxiety Depression QoL: physical QoL: mental Perceived risk Resilience Fear of progression	Prognostic testing Enucleation Graduation Age Gender	12 months Baseline younger age: *B* (SE) = −0.050 (.023), *β* = −0.147, (*p* = 0.031) Baseline higher fear of progression: *B* (SE) = 1.744 (0.524), *β* = 0.298, (*p* = 0.001) Bassline higher anxiety: *B* (SE) = 0.212 (0.102), *β* = 0.215 (*p* = 0.039) Baseline lower physical QoL: *B* (SE) = − 0.061 (0.030), *β* = −0.131 (*p* = 0.042) Baseline lower mental QoL: *B* (SE) = −0.061 (0.030), *β* = −0.178 (*p* = 0.043) *Non-significant* Graduation, resilience, social support, general distress, depression, perceived risk, enucleation and monosomy group non-significant. No significant results for fear of progression between groups. Neither the mean score for anxiety (*x*¯ = 5.38, SE = 3.84) reached the cut off for a clinically relevant condition at t4.
Melia et al. [45]	Intention to treat analysis. Markov calculation of transition probabilities between diagnostic categories across timepoints compared using log rank tests. Logistic regression used to estimate ORs at separate timepoints controlling baseline category and other covariates. Intention to treat analysis.	6 weeks after surgery, 6, 12, 24, 36, 48 and 60 months	HADS-A		Treatment modality	*Anxiety scores did not differ by treatment*.
Schuermeyer et al. [40]	Paired t-test Mixed model 2 sided and Bonferroni adjusted	Immediately pre treatment, 3 and 12 months	HADS-A	Anxiety Depression Decision Regret	Prognostication group Age Gender	Age interacted with anxiety, age predicted a decrease with older age (coefficient estimate [SE], −0.06 [0.02]; *P* < 0.001). *No association between anxiety over time and decision regret score at baseline, results of prognostication test or entry into adjuvant therapy trial*.
Van Beek et al. [44]	Multilevel hierarchical regression.	Second admission, 2, 6, 12, 24, 36 and 48 months	STAI (state trait anxiety)		Treatment modality Metastases/No metastases	36 months+ Patients with metastatic disease greater anxiety at 36 (*p* = 0.026, *d* = 0.59) and 48 months (*p* = 0.022, *d* = 0.81). *Non-significant* Treatment modality
DV – DISTRESS						
Brandberg et al. [42]	Mixed model ANOVA	First admission, 2 and 12 months	IES		Treatment modality (plaque/enucleation)	*No significant predictors*.
Lieb et al. [43]	Mixed modelling controlling age, sex and enucleation.	Hospital admission, discharge, 6–12 weeks, and 6 and 12 months.	Distress Thermometer	Social Support General distress Anxiety Depression QoL: physical QoL: mental Perceived risk Resilience Fear of progression	Prognostic testing Enucleation Graduation Age Gender	12 months Higher level of distress at baseline B (SE) 0.452 (0.105), *β* = 0.450, *p* < 0.001 *Non-significant:* age, sex, graduation, resilience, social support, fear of progression, depression, anxiety, QoL physical and mental, perceived risk, enucleation, monosomy group
Van Beek et al. [44]	Multilevel hierarchical regression. Cox regression analysis.	Second admission, 2, 6, 12, 24, 36 and 48 months	IES		Treatment modality Metastases/No metastases	Patients with mets scored slightly higher on intrusion at baseline *p* = 0.037 than those without. No sig effects in follow ups. *No difference between enucleated and fSRT patients for IES scores*.
DV – QUALITY OF LIFE						
Brandberg et al. [42]	Mixed model ANOVA.	First admission, 2 and 12 months	EORTC QLQ-C30		Treatment modality (plaque/enucleation)	*No significant differences between plaque and enucleation on QoL*.
Brown et al. [35] ^a^[linked sample]	Growth curve modelling to estimate intercepts and slopes. Intercepts and slopes predicted by prognostic variable (M3, D3, not tested, test failed)	6, 12, 24, 36, 48 and 60 months	EORTCQLQ C30 (WREC)	Chromosome 3 status	Age, Sex	M3 patients had higher worry about cancer recurrence intercepts than D3 or not tested. No difference between slopes. Thus, M3 predicts consistently higher worry about cancer recurrence than D3 or not tested. D3 did not differ from not tested.
Brown et al. [36] ^a^[linked sample]	Pearson correlations, cross-lagged analysis examining mediation of relationship between concerns about symptoms and depression by worry about cancer recurrence	6, 12, 24, 36, 48 and 60 months	EORTCQLQ C30 (WREC)	Anxiety Depression Worry about cancer recurrence	Concerns about symptoms and function (linear combination of Visual and ocular symptoms, visual function problems), Chromosome 3 status (M3 versus others), Gender, Age	Younger age predicted worry about recurrence. M3 (M3 patients vs all others) predicted worry about recurrence.
Damato et al. [37]^a^[linked sample]	Bootstrapped t-test for six month follow-up. Multilevel regression for linear trend over all observationsLinear regression.	6 months and annual observation for 10 years	EORTC QLQ-OPT30 FACT-G		Treatment modality	**6 Months** Radiotherapy was associated with fears of local recurrence (obs. coeff., 0.291) Radiotherapy less likely to experience worries about metastasis (obs. coeff., –0.164) Patients who had radiotherapy were less likely to suffer from loss of emotional well-being (obs. coeff., 0.518). **60 months**+ Enucleation late loss of **emotional well being** (obs. coeff., linear: –0.181; quadratic: –0.011)
Damato et al. [38] ^a^[linked sample]	Linear regression predicting QoL from treatment controlling treatment, pre-treatment visual acuity, eye laterality, TNM size, melanoma cytomorphology and tumour type. Linear regression predicting QoL from age, sex, time between treatment and questionnaire, general health, marital status, employment status and social support.	6 months	EORTC QLQ-OPT30 FACT-G	Self-reported QoL Social support	Treatment modality Gender Age Marital status Employment status Self-reported general health	**6 months** Radiotherapy experienced less worries about local recurrence (*p* < 0.001, OR = 0.58), metastasis (*p* < 0.001, OR = 1.49), health (*p* < 0.001, OR = 1.42, (*p* < 0.001, OR = 1.72) losing eye and appearance (*p* < 0.001, OR = 2.90) Radiotherapy better emotional well-being (*p* = 0.012, SE = 0.22). (t-test) Female sex better emotional wellbeing (*p* < 0.001, SE = 0.20). Older ager increased emotional wellbeing (*p* < 0.001, SE = 0.01). Poor social support showed lower emotional well-being (*p* < 0.001, SE = 0.08). Being employed was associated with better emotional well-being (*p* = 0.002, SE = 0.24). Better self-reported general health was associated with better emotional wellbeing (*p* < 0.001, SE = 0.09) *Non-significant* FACT-G and treatment type
Hope-Stone et al. [33] ^a^[linked sample]	Mixed model ANOVA, controlling chromosome 3 status, age, sex, health problems, logMAR (fellow eye), tumour thickness and diameter. MANOVA	6, 12 and 24 months	EORTC QLQ-OPT30 (WREC)		Treatment modality	*No significant difference for WREC*.
Hope-Stone et al. [34] ^a^[linked sample]	Mixed model multivariate ANOVA	6, 12 and 24 months	FACT-G		Treatment modality Prognosis (Monosomy 3) Age Gender	*No significant predictors*.
Lieb et al. [43]	Mixed modelling controlling age, sex and enucleation.	Hospital admission, discharge, 6–12 weeks, and 6 and 12 months.	SF-12 Fear of progression	General distress Anxiety Depression QoL: physical QoL: mental Perceived risk Resilience Fear of progression Social Support	Prognostic testing Enucleation Graduation Age Gender	*No prediction of QoL mental or fear of progression*.
Klingenstein [41]	Mixed model ANOVA	First admission, 12 and 24 months.	SF-12v2		Glaucoma Best Corrected Visual Acuity Gender	Overall Mental health improved (*p* = 0.023) after radiotherapy. Mental health increased significantly (*p* = 0.0003) in the no glaucoma group 12 months After 1 year mental health was significantly higher within the male subgroup (*p* = 0.042) but not in 2 years. 24 months Mental health was higher compared with no glaucoma patients at the second follow up (2 years) (*p* = 0.02). *Non-significant* No differences between role emotional between glaucoma groups or male vs. female group. No difference between BCVA and mental health or role emotional.
Melia et al. [45]	Generalised linear model.	6 weeks after surgery, 6, 12, 24, 36, 48 and 60 months	SF-36		Treatment modality	6 months Brachytherapy increase in mental health and emotional role function scales compared to enucleation (*p* < 0.05) however difference between both not significant. *No change for SF-36 over follow up*.
Van Beek et al. [44]	Multilevel linear regression.	Second admission, 2, 6, 12, 36, 48 months	EORTC QLQ C-30		Treatment modality Metastases/No metastases	36 months Patients with metastases showed lower emotional functioning after 36 months. (score 83.4; 95% CI, 78.4–88.4, *p* = 0.022, Student’s *t* test) *Non-significant* Treatment modality

*HADS* A Hospital Anxiety and Depression Scale, *EORTC QLQ-OPT 30* European Organisation for Research and Treatment for Cancer Ophthalmic Quality of Life Module, *SF-36* 36-Item Short Form Survey, *FACT-G* Functional Assessment of Cancer Therapy: General, *SF-12* The 12-Item Short Form Health Survey.

^a^Studies with linked sample are all from the same dataset from one research group.

### Demographic predictors

#### Gender

Findings for gender as a predictor of distress were mixed. Two out of four datasets found female gender to predict distress. In the Liverpool data set from 2008 to 2014, Brown et al. (*n* = 708) [35] consistently found higher anxiety, depression and worry about cancer recurrence in females, and greater decline of QoL in females over 5 years. This study controlled for age, chromosome 3 status and enucleation status. Using a 1993–2013 extraction from the same dataset, Damato et al. (*n* = 1596) [38] showed greater anxiety in females at 6 months after diagnosis but higher QoL (emotional wellbeing; FACT-G) at 6 months. Another dataset by Klingenstein et al. (*n* = 91) [41] found that males had significantly better QoL (mental health; SF-12) at 12 months after treatment than females, but not QoL regarding the role emotional subscale of the SF-12. This paper used the SF-12 which differentiates between mental health and role limitations due to emotional problems. No differences were found in terms of gender as a predictor of distress in two datasets for depression, anxiety (Schuermeyer et al., *n* = 96; Lieb et al., *n* = 186; *n* = 96) [40, 43], general distress or QoL (mental health; SF-12; *n* = 186) [43].

#### Age

All three datasets that investigated age found younger age to predict distress, specifically anxiety and worry about recurrence. Using the Liverpool dataset, Brown et al. (*n* = 708) [35] found initially higher anxiety and worry about recurrence over 5 years of observations in younger participants surveyed from 2008 to 2014, although the former effect decreased over time. General QoL declined over time in younger participants. This study controlled sex, chromosome 3 status and enucleation status. Additionally, using data from patients recruited from 1993–2013 using the Liverpool dataset, Damato et al. (*n* = 1596) [38] found older age predicted an increase in QoL, suggesting better scores (emotional well-being; FACT-G) at 6 months. In another dataset, Lieb et al. (*n* = 175) [43] found that younger age predicted anxiety at 12 months, but did not predict depression, general distress or QoL (SF-12). In the remaining dataset, Schuermeyer et al. (*n* = 96) [40] reported that older age predicted a decline in anxiety scores at 12 months, but not depression.

#### Marital and employment status

Only one study, using the Liverpool dataset (*n* = 1596) [38], evaluated marital and employment status as predictors of distress. Marital status did not predict distress. Being employed or a homemaker predicted lower depression scores, and better QoL (emotional well-being; FACT-G) at 6 months compared to being unemployed or retired. However, these did not predict anxiety.

### Clinical predictors

#### Treatment type

Only two out of six datasets found that treatment type—specifical enucleation compared to radiotherapy—predicted distress. The Liverpool data set found that plaque and proton beam radiotherapy treated patients suffered from less depression and less decrease in QoL (emotional well-being; FACT-G) 6 months after treatment compared to those enucleated. However, treatment differences for depression and QoL (emotional well-being, FACT-G) did not persist over the subsequent 10 years (*n* = 1596) [37]. Additionally, those enucleated also experienced a decrease in QoL (emotional well-being; FACT-G) after 24 months. In another dataset, QoL (mental health; SF-12) improved after radiotherapy (*n* = 91) [41], but no comparisons to other treatment modalities were reported. The four remaining datasets did not find any differences for treatment type as a predictor of depression, (Brandberg et al., *n* = 99; Lieb et al., *n* = 186; Melia et al., *n* = 209) [42, 43, 45] anxiety (Brandberg et al., *n* = 99; Lieb et al., *n* = 186; Melia et al., *n* = 209; van Beek et al., *n* = 113) [42–45], emotional distress (Brandberg et al., *n* = 99; Lieb et al., *n* = 186; van Beek et al., *n* = 113) [42–44] or QoL (Brandberg et al., *n* = 99; Lieb et al., *n* = 186; Melia et al., *n* = 209; van Beek et al., *n* = 113) [42–45].

#### Chromosome 3

Only one out of the three data sets that accounted for M3 status predicted emotional distress. In the Liverpool dataset, Brown et al. (*n* = 708, recruited 2008–2014) [35] found that M3 survivors (compared to D3 and those who had either opted not to be tested, or whose results were inconclusive) had consistently higher depression, anxiety and worry about recurrence scores over the 5 years, than non-M3 patients. Another paper by Damato et al. (*n* = 1596) [38], extracted from the Liverpool dataset with participants recruited from 1993 to 2013, reported that the presence of M3 predicted higher levels of anxiety. The two remaining datasets did not find any prediction between chromosome status and anxiety, depression (Lieb et al., *n* = 186; Schuermeyer et al., *n* = 96) [40, 43], QoL or distress (Lieb et al., *n* = 186) [43].

#### Physical health predictors

All three datasets found that physical health variables, including physical symptoms and functional difficulties associated with UM, predicted emotional distress. The Liverpool dataset found that self-reported concerns about visual and ocular symptoms and functional impairments (these variables were reduced by confirmatory factor analysis to a single predictor variable) and 6-month visual, ocular and impairment concerns predicted 12 month anxiety, 24-month visual, ocular and impairment concerns predicted 36 month anxiety, and 48 month visual and ocular concerns predicted 60 month anxiety (Brown et al., *n* = 453, recruited 2008–2014) [36]. In a more fine-grained analysis, using a subset of the data used by Brown et al. (*n* = 703) [35], Brown et al. (*n* = 291) [32] found that self-reported 6-month ocular irritation, headaches and functional impairment predicted anxiety, and headaches and functional impairment predicted depression, at 24 months. Better self-reported general health predicted lower anxiety and depression, in addition to better QoL (emotional wellbeing; FACT-G) (Damato et al., *n* = 1596, recruited 1993–2013) [38].

Using data from clinical records, Brown et al. (*n* = 453) [36] found that visual acuity did not predict distress at any time point. In another dataset, for patients who did not develop secondary glaucoma, QoL (mental health) improved at 24 months compared to those who did not (*n* = 91) [41]. However, this was not true for another subscale of QoL (role emotional; SF-12). Both subscales are part of the SF-12, which differentiates between mental health and role limitations due to emotional problems. In the remaining dataset, van Beek et al. (*n* = 113) [44] found that the development of metastatic disease significantly predicted anxiety after 3 years, but did not predict general distress or QoL.

### Social predictors

Only two datasets investigated social support as a predictor of emotional distress. One from the Liverpool dataset found that better social support predicted a decrease in anxiety and depression, and better QoL (emotional well-being; FACT-G) at 6 months (*n* = 1596) [38]. No significant predictors were found in the other dataset of anxiety, depression, distress or QoL (*n* = 186) [43].

### Psychological predictors

#### Emotional distress

All three studies, drawing from three datasets, that examined whether measures of distress predicted subsequent distress found significant findings. Lieb et al. (*n* = 175) [43] found that general distress at 12 months was predicted only by elevated scores of general distress at admission to hospital (immediately pre-treatment), but not depression, anxiety or QoL. Twelve-month depression was predicted by lower physical quality of life and high depression scores at admission to hospital (immediately pre-treatment), but not general distress, QoL or anxiety. Twelve-month anxiety was predicted by, high baseline anxiety scores and lower physical and mental quality of life, but not depression, resilience, or general distress. Additionally, Schuermeyer et al. (*n* = 91) [40] found that 12-month depression, increased with baseline anxiety and anxiety increased with depression scores at admission to hospital (immediately pre-treatment), however anxiety scores were higher at admission to hospital (immediately pre-treatment), than all three follow up points. Brown et al.’s (*n* = 291) [32] analysis of data from the Liverpool dataset found that 6- and 12-month anxiety and depression predicted both 24-month anxiety and depression caseness.

#### Worry about recurrent disease and fear of progression

Two studies, using data from two datasets, considered whether worry about recurrent disease and fear of progression predicted distress. Lieb et al. (*n* = 186) [43] found that higher fear of progression predicted 12-month depression and anxiety, but not general distress. Analysing data from the Liverpool dataset using cross-lagged analyses, Brown et al. (*n* = 453) [36] reported that worry about recurrent disease at 12 months post-diagnosis predicted anxiety at 24 months, worry about recurrent disease at 48 months predicted anxiety at 60 months in females, and worry about recurrent disease at 24 months predicted anxiety at 48 months in males. Additionally, worry about recurrent disease at 24 and 36 months predicted depression at 36 and 48 months, respectively.

#### Resilience

Lieb et al. (*n* = 186) [43] considered resilience and found it predicted depression at 12 months, but did not predict general distress or anxiety.

#### Decision regret

One study considered whether decision regret about whether to undergo prognostic testing for chromosome 3 loss, predicted distress and found no prediction for depression or anxiety (*n* = 91) [40].

## Discussion

This review is the first to examine only prospective studies to identify demographic, clinical, social and predictors of emotional distress in UM survivors. Thirteen papers were identified, seven of which used an overlapping dataset, resulting in seven distinct datasets. Overall, the most consistent predictors of distress were younger age, physical health problems; self-reported visual and ocular symptoms, functional impairments, and clinically reported secondary glaucoma and metastatic disease and psychological predictors, namely baseline distress and worry about recurrence.

For demographic predictors, younger age predicted distress, specifically elevated anxiety, in all datasets that examined it. This is consistent with research on other cancers [2, 46, 47]. Younger people may experience greater distress because they perceive unfairness where a diagnosis is either less common or implies greater loss of life years [48]. This can lead to a perceived threat to life goals, which is a key factor in adjusting to chronic conditions [49]. Younger patients may also perceive greater disruption to social roles including work and family [2]. However, younger age was only found to consistently predict anxiety but no other measures of distress, including depression or lower subscales of QoL. As mixed findings were found for gender, with some evidence to suggest that females are at greater risk of distress, this may be attributable to higher level of reporting distress among females [50]. These specific populations may need additional screening and psychological support to prevent and alleviate distress in all datasets that examined it.

Only two out of six datasets that investigated this variable, found that treatment type predicted distress, although the largest study showed that enucleation yielded worse outcomes compared to other treatments. These results are consistent with cross-sectional, retrospective and follow-up studies suggesting that treatment type is not reliably associated with emotional distress [11, 30]. Studies with larger samples found that enucleation and M3 status predicted subsequent distress, although these were not detected in studies with smaller samples, possibly because effect sizes were small. The inconsistency between treatment effects may be attributable to differential trajectories of adverse outcomes for enucleation and proton beam radiotherapy. Enucleation initially reduces visual functioning which improves over time, whereas visual symptoms and functioning can gradually worsen after radiotherapy [33]. Assuming that distress is contingent on heightened symptoms and functional problems [36], treatment differences are likely to vary over time.

A key paper with a large sample and 5-year follow up found M3 status to be associated with greater distress, although this finding is qualified as distress did not significantly exceed community norms [35]. Other studies found no association between chromosome 3 status and distress, comparable with a previous systematic review which included several cross-sectional and retrospective studies, where no consistent associations were found [11]. Intuitively, it may be surprising that reliable life expectancy prognostication, with the capacity to deliver news of severely limited or almost normal life expectancy estimates, has limited impact on distress. One possibility is that patients muted the impact of estimates. One study showed that patients remained uncertain when given prognostic estimates with those receiving good prognoses failing to trust them, and those reporting poor prognoses finding alternative ways of building hope for the future [51].

Less surprisingly, poorer physical health, presence of physical symptoms and functional difficulties predicted emotional distress. First, self-reported treatment-related visual and ocular symptoms and functional impairments predicted greater distress. This was mediated by increases in worry about recurrence, suggesting that the presence of symptoms in the treated eye might either be mistaken for local recurrence or symptom similarity may trigger patients’ memories of previous diagnosis of cancer [52]. Second, clinical health-related QoL, metastatic disease and glaucoma were related to emotional distress. This is consistent with a wealth of research showing emotional distress attributable to physical illness [53]. The findings highlight the importance of ensuring that symptoms and concerns about health are carefully managed as part of patients’ health care plan.

Social predictors were only investigated in two of seven datasets, with conflicting findings in relation to whether social support predicted distress. Only one study considered demographic factors such as marital or employment status with being in employment predicting less distress and increased wellbeing [38]. Social factors such as socio-economic status and availability of social resources impact how a person appraises, adjusts, and copes with chronic conditions, which influence levels of emotional distress and physical health [54, 55]. Further research could investigate the relationship between these factors and distress in UM.

In terms of psychological predictors, a consistent finding was that initial distress scores during the first data measurements predicted future distress in three out of three datasets. These findings are in keeping with previous literature reviews [6] which found that baseline distress consistently predicted emotional distress in cancer survivors. It is unclear whether these findings reflect distress that is attributable to UM or whether distress was present prior to diagnosis. Furthermore, it should be considered that half of the papers measured the first data measurement at 6 months, compared to the remaining papers that examine data around the time of diagnosis. Nonetheless, this finding provides insight into how those who display initially high levels of distress may be at greater risk of distress over time, however it must be considered that distress at initial stages of a cancer diagnosis is to be expected and often resolves without intervention over time [56]. Moreover, these findings highlight the importance of routine psychological assessments early in the cancer trajectory, as recommended by clinical guidelines for cancer care [57]. Psychological processes such as worry about recurrence were significant in two out of two datasets, in addition to a single study suggesting that lower resilience predicted distress. Very few studies examined these and other psychological processes, such as coping behaviour, hopelessness and neuroticism however these have been found to predict distress when considering cancer more generally [7]. Further exploration of psychological constructs such as resilience and worry, which are modifiable through psychological intervention is imperative to inform the design and implementation of such support [58, 59]. No research to date has considered the efficacy of psychological intervention for UM patients.

### Strengths and limitations

This review considered prospective studies only, which provide a stronger evidence base than cross-sectional data for drawing conclusions regarding factors which predict distress in UM. The variables assessed did not consistently find that distress was predicted at comparable time points, therefore it has not been possible to determine whether people are at increased or decreased risk of elevated distress after a certain amount of time. Many papers only had follow up of a year or two years after diagnosis. Emotional distress was investigated using validated measures of anxiety and depression, predominately the HADS [60]. Patients with UM previously assessed using the HADS displayed non-clinical levels of anxiety and/or depression however were identified by a psychologist in clinical practice as needing psychological intervention and vice versa [61]. This suggests that distress levels from the HADS is not the only important issue to screen to help inform clinical decision making about whether a patient may need psychological intervention. Furthermore, the questionnaires used by studies in this review included the use of subscales for the measurement of QoL e.g., emotional and mental health subscales. This raises questions about whether robust conclusions can be drawn from the outcome of the data as subscales may not fully encapsulate distress, as the measures rely on fewer statements for theoretical validity. This could explain why some datasets found significant results for one subscale of QoL but not the other, such as role emotional and mental health. However, the use of validated subscales permits a closer examination of important clinical dimensions which would otherwise not be captured.

There is a need for further research into the predictors of distress in those diagnosed with UM as the current evidence is limited. The quality of the papers included in this review was all adequate in the categories assessed for sampling and follow up duration. One area where quality could be strengthened in future research is in attrition rates and reporting. Larger samples with broader investigations into potential predictors with a prospective design are recommended.

The findings of this review highlight areas where attention could be directed for preventing and alleviating distress in UM. Ophthalmologists and ocular oncologists can play a key role in supporting their patients with a cancer diagnosis [62]. In line with guidelines for cancer care, health care professionals are recommended to recognise and pay attention to psychological needs, and, if it seems appropriate, refer on for further psychological assessment to determine if patients might benefit from intervention to reduce psychological distress [57]. Psychological screening and intervention early on in the cancer trajectory can positively influence the well-being of patients [63]. It is important to consider the potential role of physical health and functional difficulties, younger age and distress early in a cancer diagnosis and how these may heighten the need for supportive care.

## Conclusion

Limited prospective research has been conducted to investigate what factors predict and maintain emotional distress in UM. Research has predominantly considered physical and clinical aspects of UM such as treatment type, M3 status and demographic predictors. Inconsistencies were found for treatment type, gender and M3 in predicting emotional distress. Age consistently predicted anxiety and could therefore be part of screening for distress in this population. Physical predictors such as self-reported ocular and functional difficulties and clinical outcomes of metastatic disease and secondary glaucoma were also found to be significant predictors. Additionally, psychological predictors such as baseline distress and worry about recurrence were considered in fewer studies, however they consistently predicted emotional distress and highlight initial promising findings. There is a need to explore the role of other psychological factors in predicting distress. Possibilities include resilience, neuroticism and coping behaviour which have been found in other tumour groups [7]. This review highlights the need for careful consideration for management of physical or functional concerns in UM to help prevent long term emotional distress.
